# Hydrogen sulfide increases excitability through suppression of sustained potassium channel currents of rat trigeminal ganglion neurons

**DOI:** 10.1186/1744-8069-9-4

**Published:** 2013-02-18

**Authors:** Xingmei Feng, You-Lang Zhou, Xiaowen Meng, Fei-Hu Qi, Wei Chen, Xinghong Jiang, Guang-Yin Xu

**Affiliations:** 1 Department of Stomatology, Affiliated Hospital of Nantong University, Nantong 226001, China; 2 Department of Neurobiology, Institute of Neuroscience, Key Laboratory of Pain Research & Therapy, Soochow University, Suzhou 215123, China; 3 Jiangsu Key Laboratory of Neuroregeneration, Nantong University, Nantong 226001, China

**Keywords:** Hydrogen sulfide, Cystathionine-β-synthase, Trigeminal ganglion, Excitability, Voltage-gated potassium channels

## Abstract

**Background:**

Hydrogen sulfide (H_2_S), an endogenous gaseotransmitter/modulator, is becoming appreciated that it may be involved in a wide variety of processes including inflammation and nociception. However, the role and mechanism for H_2_S in nociceptive processing in trigeminal ganglion (TG) neuron remains unknown. The aim of this study is to investigate distribution of endogenous H_2_S synthesizing enzyme cystathionine-β-synthetase (CBS) expression and role of H_2_S on excitability and voltage-gated potassium channels of TG neurons.

**Methods:**

Immunofluorescence studies were carried out to determine whether CBS was co-expressed in Kv1.1 or Kv1.4-positive TG neurons. Whole cell patch clamp recordings were employed on acutely isolated TG neurons from adult male Sprague Dawley rats (6–8 week old). von Frey filaments were used to examine the pain behavioral responses in rats following injection of sodium hydrosulfide.

**Results:**

In rat TG, 77.3±6.6% neurons were immunoreactive for CBS, 85.1±3.8% for Kv1.1 and 97.8±1.1% for Kv1.4. Double staining showed that all CBS labeled cells were Kv1.1 and Kv1.4 positive, but only 92.2±6.1% of Kv1.1 and 78.2±9.9% of Kv1.4 positive cells contained CBS. Application of H_2_S donor NaHS (250 μM) led to a significant depolarization of resting membrane potential recorded from TG neurons. NaHS application also resulted in a dramatic reduction in rheobase, hyperpolarization of action potential threshold, and a significant increase in the number of action potentials evoked at 2X and 3X rheobase stimulation. Under voltage-clamp conditions, TG neurons exhibited transient A-type (*I*_A_) and sustained outward rectifier K^+^ currents (*I*_K_). Application of NaHS did suppress *I*_K_ density while did not change *I*_A_ density of TG neurons (n=6). Furthermore, NaHS, a donor of hydrogen sulfide, produced a significant reduction in escape threshold in a dose dependent manner.

**Conclusion:**

These data suggest that endogenous H_2_S generating enzyme CBS was co-localized well with Kv1.1 and Kv1.4 in TG neurons and that H_2_S produces the mechanic pain and increases neuronal excitability, which might be largely mediated by suppressing *I*_K_ density, thus identifying for the first time a specific molecular mechanism underlying pain and sensitization in TG.

## Introduction

Hydrogen sulfide (H_2_S), a gas synthesized by the endogenous enzymes cystathionine-β-synthetase (CBS) and cystathionine-γ-lyase (CSE), is increasingly recognized as a biologically important signaling molecule in various tissues and pathophysiological processes including pain and inflammation [1-7]. Its putative role as a neurotransmitter is supported by recent reports on its effects on hippocampal neurons as well as peripheral sensory neurons [7-9]. With respect to the latter, intraplantar injection of NaHS (a commonly used H_2_S donor) in rat hindpaws produces mechanical hyperalgesia through activation of T-type Ca^2+^ channels [8], supporting a pro-nociceptive role for H_2_S. Further, H_2_S generation is also enhanced in formalin [9] and carrageenan [10] model of persistent inflammatory pain. Colonic administration of H_2_S enhances pain behaviors in response to CRD in mice [3] and rats [11]. On the other hand, systemic injections of H_2_S donors in rats suppress responses to colorectal distention (CRD) by activating K_ATP_ channels [12], suggesting a possible anti-nociceptive effect. Although there is discrepancy, a growing body of evidence suggests that H_2_S plays an important effect on primary sensory neurons innervating somatic and visceral organs. However, the role of H_2_S on trigeminal ganglion (TG) neurons in the normal neuronal physiology remains unknown.

The aims of the present study were therefore to determine distribution of endogenous H_2_S synthyzing enzymes in TG neurons and to identify roles of H_2_S on neuronal excitability by brief exposure to NaHS, at concentrations near the reported K_D_ values in other cellular systems. We also examined NaHS’s influence on the delayed-rectifier type of K^+^ currents, since such currents are an important factor in the ability of cells to fire impulses repetitively and have been identified as important parameters for the modulation of excitability by other agents, such as prostaglandin E_2_[13] and nerve growth factor [14]. Potassium channels also have been identified as targets of drugs that relieve elevated pain. Our results show that CBS is abundantly expressed in rat TG neurons and colocalized in Kv1.1 and Kv1.4 positive neurons. H_2_S enhances excitability of TG neurons and suppresses *I*_K_ density *in vitro* and reduces the escape threshold of rats.

## Methods

### Animals

Experiments were performed on adult male Sprague-Dawley rats (200–220 g). Care and handling of these animals were approved by the Institutional Animal Care and Use Committee at Soochow University and were in accordance with the guidelines of the International Association for the Study of Pain.

### Immunofluorescence study

Male adult rats were deeply anesthetized with sodium pentobarbital (50 mg/kg, i.p.) and perfused transcardially with 150 mL phosphate-buffered saline (PBS) followed by 400 mL ice-cold 4% paraformaldehyde (PFA) in PBS. A pair of trigeminal ganglion was then excised and postfixed in this fixative solution and cryoprotected overnight in 20% sucrose in PBS. Ten-micrometer sections were cut in a cryostat and processed for immunofluorescence as described previously [15]. In brief, all sections were blocked with 2% goat serum and incubated overnight at 4°C with primary antibody, followed by TRITC or FITC-conjugated secondary antibody. For double immunofluorescence, sections were simultaneously incubated with CBS (1:200, Abnova, CA) and Kv1.1 or Kv1.4 antibody (1:200, Alomone labs, Israel) and then incubated with TRITC and FITC-conjugated secondary antibody. Negative control was performed by omitting the primary antibody. Images were captured and analyzed using Metaview software (Universal Imaging Corporation Nikon, Melville, NY). To ensure that a neuron was counted only once, serial sections were placed on consecutive slides with at least 50 μm between sections on the same slide.

### DiI labeling

Temporomandibular joint (TMJ) receptive field specific TG neurons were labeled by injection of 1,19-dioleyl-3,3,39,3-tetramethy-lindocarbocyanine methanesulfonate (DiI; Invitrogen, Carlsbad, California) as described previously [16]. In brief, animals were anesthetized with a cocktail of ketamine (80 mg/kg) and xylazine (5-10 mg/kg, intraperitoneally). The skin overlying the TMJ was shaved. The injection site was identified by palpating the zygomatic arch and mandible. DiI (25 mg in 0.5 ml methanol) was injected into both sides of TMJ skin (1 μl/site, 3 sites in each side). Multiple small injections of the tracers were made to limit the spread of the tracer into untargeted tissues [17]. To prevent leakage, needle was left in place for 1 min for each injection. Ten days later, both TGs were dissected out for patch clamp recordings. The appearance of the injected tracer in TG neurons indicates their innervation zone in the overlying skin.

### Dissociation of TG neurons

Isolation of TG neurons from adult male rats has been described previously [18,19].

Briefly, animals were killed by cervical dislocation, followed by decapitation. A pair of TGs were then bilaterally dissected out and transferred to an ice-cold, oxygenated fresh dissecting solution, which contained (in mM): 130 NaCl, 5 KCl, 2 KH_2_PO_4_, 1.5 CaCl_2_, 6 MgSO_4_, 10 glucose, and 10 HEPES, pH 7.2 (osmolarity: 305 mOsm). After removal of connective tissue, ganglia were transferred to 5 ml of dissecting solution containing collagenase D (1.8 –2.0 mg/ml, Roche; Indianapolis, IN) and trypsin (1.2 mg/ml, Sigma; St. Louis, MO) and incubated for 1.5 h at 34.5°C. TGs were then taken from the enzyme solution, washed, and transferred to 2 ml of the dissecting solution containing DNase (0.5 mg/ml, Sigma, St. Louis, MO). A single-cell suspension was subsequently obtained by repeated trituration through flame-polished glass pipettes. Cells were plated onto acid-cleaned glass coverslips.

### Patch-clamp recordings

Coverslips containing adherent TG cells were put in a small recording chamber (0.5 ml volume) and attached to the stage of an inverting microscope (Olympus). For patch-clamp recording experiments, cells were continuously superfused (1.5 ml/ min) at room temperature with normal external solution containing (in mM) 130 NaCl, 5 KCl, 2 KH_2_PO_4_, 2.5 CaCl_2_, 1 MgCl_2_, 10 HEPES, and 10 glucose, with pH adjusted to 7.4 with NaOH (osmolarity: 295-300 mOsm). DiI-labeled neurons were identified by the bright red fluorescence in the cytoplasm. Recording pipettes were pulled from borosilicate glass tubing using a horizontal puller (P-97, Sutter Instruments) and typically had a resistance of 3.5-4.5 M when filled with normal external solution before being used immediately to obtain a gigaohm seal. Tip potential was zeroed before membrane-pipette seals were formed. The voltage was clamped at −60 mV by an EPC10 amplifier (HEKA, Germany). Capacitive transients were corrected using capacitive cancellation circuitry on the amplifier that yielded the whole cell capacitance and access resistance. Up to 80% of the series resistance was compensated electronically. Considering the peak outward current amplitudes of 10 nA, the estimated voltage errors from the uncompensated series resistance would be 10 mV. The leak currents at −60 mV were always below 20 pA and were not corrected. The action potentials and potassium currents were filtered at 2–5 kHz and sampled at 50 or 100 μs/point. Data were acquired and stored on a Dell computer for later analysis using Patch Master (HEKA, Germany). All experiments were performed at room temperature (~22°C). Only neurons with a stable initial resting potential, which drifted by less than 2–3 mV during the 10 min of baseline recording, were used in these experiments. Cells were characterized by their resting potential, input resistance, Rm. Stimulating ramps of linearly increasing current (range 0.2–0.5 nA/s) were chosen to produce more than action potentials (APs) over a 1 s depolarization for each tested neuron before and after NaHS application. In addition to the number of APs during the ramp, the AP threshold.

### Isolation of Kv currents

To record K_V_ currents, Na^+^ in control external solution was replaced with equimolar choline and Ca^2+^ concentration was reduced to 0.03 mM to suppress Ca^2+^ currents and to prevent Ca^2+^ channels becoming Na^+^ conducting. The reduced external Ca^2+^ would also be expected to suppress Ca^2+^-activated K^+^ current [15]. The following two kinetically distinct Kv currents were isolated by the biophysical analysis and pharmacological approaches described in previous studies: *I*_A_ and *I*_K_[20,21]. *I*_A_ and *I*_K_ were separated biophysically by manipulating the holding potentials. The total outward currents (*I*_Total_) were recorded in response to voltage steps from −100 to +30 mV in 5-mV increments with duration of 400 ms. *I*_K_ was isolated when the membrane potential was held at −50 mV. Subtraction of *I*_K_ from *I*_Total_ represented *I*_A_. To control for changes in cell size, the current density (pA/pF) was measured by dividing the current amplitude by whole cell membrane capacitance, which was obtained by reading the value for whole cell input capacitance cancellation directly from the patch-clamp amplifier.

### Drug application

NaHS was purchased from Sigma-Aldrich (St. Louis, MO) and was freshly prepared in normal external solution for observing RP and APs under current clamp conditions or the external solution for recording K^+^ current under voltage clamp conditions as described above. NaHS (250 μM) was applied directly to the recorded cell by pressure and therefore could reach equilibrium almost instantaneously. For behavioral study, NaHS (50, 250 and 1000 μM) in 50 μL was injected into the right side of the temporomandibular joint capsule, which was identified by palpating the zygomatic arch and condyle [22]. A 30-gauge needle was inserted into the point immediately inferior to the posteroinferior border of the zygomatic arch. The needle was advanced in an anterior direction until it contacted the posterolateral aspect of the condyle. An equal volume of normal saline (NS) was used as control.

### Mechanical threshold for escape behavior

Mechanical hyperalgesia was measured by a previously described protocol with some minor modifications [22,23]. Rats were weighed and placed individually in small plastic cages and allowed to adapt to the observation cage and to the testing environment for over 1 h. During this period, the experimenter reached slowly into the cage to touch the walls of the cage with a plastic rod. After the rats were habituated to the reaching movements, the series of mechanical stimulations were started. The virissas were carefully shaved and the ipsilateral and contralateral facial skin regions were tested. The mechanical response threshold of escape behavior was measured before and after injection of NaHS or NS. A graded series of von Frey filaments were used. The filaments produced a bending force of 0.55, 0.93, 1.61, 1.98, 2.74, 4.87, 7.37, 11.42, 15.76, 20.30, and 38.69 g. The response was observed to belong to one or one more of the following responses: 1) the rat turns the head slowly away or moves it briskly backward when the stimulation is applied, and sometimes a single face wipe ipsilateral to the stimulated area occurs; 2) the rat avoids further contact with the stimulus object, either passively by moving its body away from the stimulating object to assume a crouching position against the cage wall, or actively by attacking the stimulus object, making biting and grabbing movements; 3) the rat displays an uninterrupted series of at least three face-wash strokes directed toward the stimulated facial area. Each filament was tested three times at an interval of 30 seconds. If response was observed at least two times after probing with a filament, the rats were considered responsive to that filament. The response threshold was defined as the minimum intensity for eliciting the response according to the method described by Chaplan and collaborator [24].

### Data analysis

All values are given as mean±SEM. No neuron with a resting membrane potential more depolarized than −40 mV was included in the data analysis. Statistical significance was determined by one-way ANOVA or Student’s *t*-test or Fisher’s exact test, as appropriate. p<0.05 was considered statistically significant.

## Results

### CBS is abundantly expressed by TG neurons

**Figure 1:**
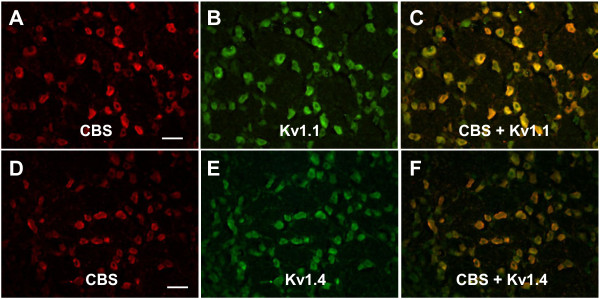
**CBS expression in trigeminal ganglion (TG) neurons.** CBS-like immunoreactivity (CBS-LI) was observed in TG cells. Note that most of the small and medium sized cells were CBS-LI positive and large cells were weakly labeled or negative (**A** and **D**). TG cells were labeled with CBS (red). **B** and **E**, expressions of Kv1.1 (**B**) and Kv1.4 (**E**) in TG neurons were shown in green. **C** and **F**, Merge of double labelings of CBS with Kv1.1 (**C**) or Kv1.4 (**F**). Bar=100 μm.

We first examined the endogenous H_2_S producing enzyme CBS expression in TG neurons by immunohistochemistry. CBS-like immunoreactivity (CBS-LI) was, on average, present in 77.0±6.6% of TG neurons (n=3 rats). CBS-LI was predominantly expressed in small and medium-size neurons, with large DRG neurons staining weakly or negative (Figure 1A and D). The expression of the other H_2_S producing enzyme CSE was also determined. In contrast to the findings of CBS, the CSE antibodies did not reveal reliable and reproducible staining in rat TG neurons and quantitative assessment was therefore not feasible.

### CBS is co-localized with Kv1.1 and Kv1.4 in TG neurons

We next examined whether CBS was co-expressed in Kv1.1 or Kv1.4-positive TG neurons since these two molecules are involved in transduction of nociceptive information in TG. Double labeling studies were performed in this experiment. Trigeminal ganglion sections were stained with CBS and Kv1.1 (Figure 1B) or Kv1.4 antibodies (Figure 1E). In normal rats (n=3), 85.1±3.8% TG neurons were immunoreactive for Kv1.1 and 97.8±1.1% for Kv1.4. Almost all the CBS labeled cells were Kv1.1 and Kv1.4 positive, but only 92.2 ± 6.1% and 78.2 ± 9.9% of Kv1.1 and Kv1.4 positive cells contained CBS-LI, respectively (Table 1).

**Table 1:** **Distribution of CBS and K**_**v**_**1.1 or K**_**v**_**1.4 in TG neurons**

**% of CBS position neurons**	**% of K**_**v**_**1.1 position neurons**	**% of K**_**v**_**1.4 position neurons**
77.3±6.6	85.1±3.8	97.8±1.1
% of K_v_1.1 position neurons		% of K_v_1.4 position neurons
Containing CBS		Containing CBS
92.2±6.1		78.2±9.9

Values are means±SE; n=3 rats.

### NaHS depolarizes resting membrane potentials

**Figure 2:**
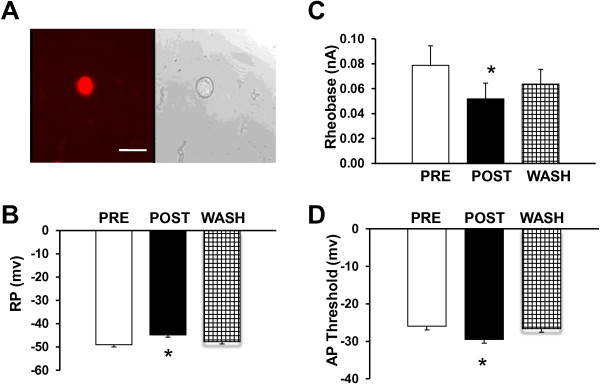
**Enhanced excitability by NaHS application. A**, representative photographs of DiI-label TG neurons (red, left) and differential interference contrast image (DIC, right). Bar=50 μm. **B**, NaHS, a donor of H_2_S at 250 μM was perfused for 3 min. NaHS application significantly depolarized the resting membrane potentials (RP, n=11). **C**, NaHS application markedly reduced rheobase (n=11). **D**, NaHS application significantly increased action potential (AP) threshold. Of note is that the effects of NaHS on neuronal excitability were reversible. Values show mean ± S.E.M. *p<0.05 (n=11) versus PRE.

To determine the effect of H_2_S on excitability of TG neurons, resting membrane potentials (RPs) of TG neurons were first studied. TG neurons were acutely dissociated and DiI-labeled neurons were studied using current-clamp techniques (Figure 2A). Before application of NaHS donor for H_2_S, RPs of neurons recorded were very stable within 2 min of observation, and no spontaneous firing was seen. The average RP was −49.1 ±1.3 mV (n=11). After application of NaHS (250 μM) for 3 min, RPs of neurons recorded showed marked depolarization. The average RP was −44.8 ±1.0 mV (n=11) after NaHS application (*p*<0.05, when compared with PRE, Figure 2B). The effect of NaHS on RP was revisable. The RP was -47.7±3.6 mV (n=11) after wash. Application of NaHS did not significantly alter the cell size and membrane input resistance of TG neurons (data not shown).

### NaHS increases excitability of TG neurons

**Figure 3:**
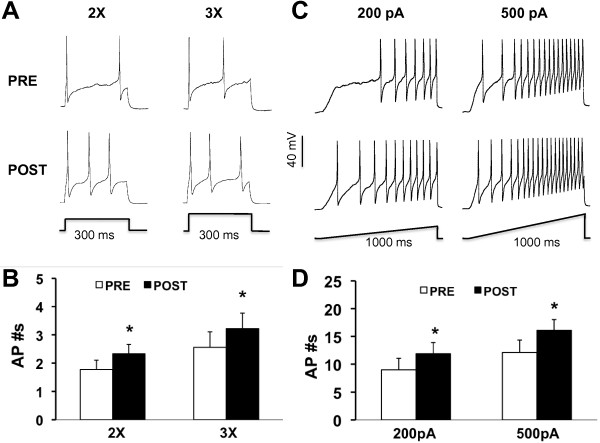
**Increase in numbers of action potentials after NaHS application. A**, action potentials were induced in TG neurons by a 2X (120 pA) and 3X (180 pA) rheobase current injection in the absence (top) or presence (bottom) of 250 μM NaHS. **B**, Bar graph showed significant increase in numbers of APs after NaHS application. **C**, APs (upper traces) were induced by a ramp current injection (lower traces, 200 pA, left; 500 pA, right) in the absence (top) and presence (bottom) of 250 μM NaHS. **D**, Bar graph showed a significant increase in numbers of APs in response to ramp stimulation after NaHS application. * p<0.05 (n=6) versus control (PRE).

To further study alteration of neuronal excitability after NaHS application, we next examined active membrane properties including current threshold (rheobase), AP threshold, and pattern of firings in response to depolarizing current stimulation. The rheobase is the minimal current injection to induce one action potential (AP). In this study, the average rheobase of TG neurons was markedly lower after NaHS incubation than pre-NaHS application (Figure 2*C*, *p*<0.05). AP threshold is the minimal voltage at which the AP was generated. NaHS incubation led to a significant increase in AP threshold of TG neurons (Figure 2D). The average AP threshold was −26.0 ±1.3 mV and -29.5±1.4 mV (n=11) before and after NaHS application, respectively (*p*<0.05, when compared with PRE, Figure 2B). The effect of NaHS on AP threshold was revisable. The AP threshold was −26.6±2.2 mV (n=11) after wash. In addition, the numbers of APs in response to a current stimulation (2X, 3X rheobase, and ramp) were remarkably increased after 3 min NaHS incubation. The number of AP numbers in response to 2X and 3X time current stimulation before NaHS application was 1.8±0.3 APs/300 ms (*n*=6) and 2.6±0.7 APs/300, respectively. After NaHS applicaton, the number of AP numbers in response to 2X and 3X time current stimulation before NaHS application was 2.4±0.3 APs/300 ms (*n*=6) and 3.1±0.8 APs/300, respectively (Figure 3A &B, *p<0.05). However, AP duration at 0 mV, amplitude and overshoot were not significantly altered after NaHS application (data not shown).

To further compare numbers of AP firing of TG neurons before and after NaHS application, we also used 1 second ramp current injections from 0 pA to 200 pA or 500 pA (Figure 3C & D). Because APs elicited by ramp current injection showed adaptation in some neurons, we counted only overshooting APs (i.e., AP with peak >0 mV). The average numbers of APs before NaHS application were 9.0±2.1 and 12.1±2.2 for 200 pA and 500 pA, respectively. After NaHS (250 μM) application, the average numbers of APs were 11.9±2.0 and 16.1±1.9 for 200 pA and 500 pA, respectively. The use of NaHS significantly enhanced the number of APs evoked by 200 or 500 pA current injection.

### NaHS suppresses I_K_ density of TG neurons

**Figure 4:**
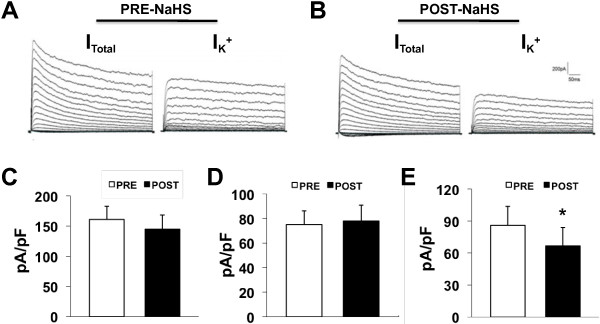
**Effects of NaHS on *I***_**A **_**and *I***_**K**_**.** Currents were measured at different holding potentials. For total voltage-gated K current, the membrane potential was held at -100 mV and voltage steps were from -40 to +30 mV with 5-mV increments and 400-ms duration. For sustained Kv current (*I*_k_), the membrane potential was held at -50 mV and the voltage steps were the same as above. Currents generated by these two protocols were subtracted to produce *I*_A_. **A**, An example of *I*_Total_ (left) and *I*_K_ (right) of a DiI labeled TG neurons before application of NaHS. **B**, An example of *I*_Total_ (left) and *I*_K_ (right) of the same TG neuron in Figure 4B 3 mins after application of NaHS. Bar graphs showed average of the peak current densities of *I*_Total_ (**C**), *I*_A_ (**D**) and *I*_K_ (**E**) currents. The current density (in pA/pF) was calculated by dividing the peak current amplitude by cell membrane capacitance. NaHS application caused a significant reduction of *I*_K_. Current subtraction revealed that NaHS application did not alter the *I*_A_ density. *p<0.05, compared with that of PRE.

Because changes in spike frequency and activation thresholds suggest an alteration in Kv channels, we next performed patch-clamp recordings to examine these currents under voltage-clamp conditions. Na^+^ in the control external solution was replaced with equimolar choline and the Ca^2+^ concentration was reduced to 0.03 mM, as described previously [25-27]. A depolarization step from -100 to +30 mV in 5-mV increments with duration of 400 ms activated all Kv channels (*I*_Total_; Figure 4A and 4B left). NaHS treatment did not significantly altered *I*_total_ density in TG neurons compared with that before NaHS application (PRE: 161.0±21.9 pA/pF; POST: 145.4±23.5 pA/pF, *n*=6, *p*<0.05; Figure 4C). Because there were two main types of Kv currents (*I*_A_ and *I*_K_) described in nociceptive TG neurons, we then isolated these two kinetically different Kv currents by manipulating the holding membrane potential. A depolarization step from -50 to +30 mV in 5-mV increments with duration of 400 ms activated most of the sustained Kv channels but not A-type Kv channels (Figure 4A and B right). Subtraction of *I*_K_ from *I*_Total_ yields *I*_A_. *I*_A_ was further confirmed by the application of the A-type channel blocker 4-aminopyridine (4-AP; 5 mM, data not shown). In this experiment, *I*_K_ density was remarkably reduced after NaHS application (PRE: 85.9±17.9pA/pF; POST: 66.6±17.3pA/pF, n=6, *p*<0.05; Figure 4E), whereas *I*_A_ density was not significantly changed (PRE: 75.7±11.2 pA/pF; POST: 78.4±12.9 pA/pF, *n*=6, *p*>0.05; Figure 4D).

### NaHS produces mechanical hyperalgesia

**Figure 5:**
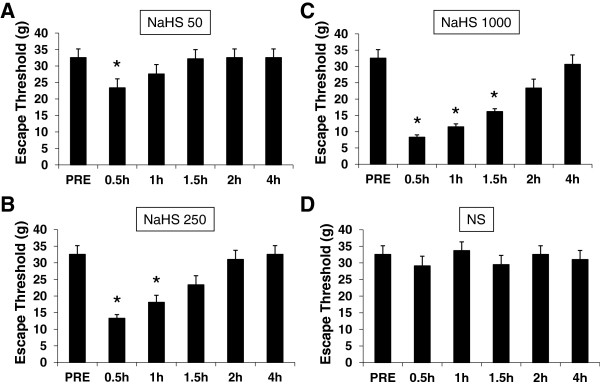
**Reduction in escape threshold by NaHS injection.** NaHS, a donor of H2S, produced mechanical hyperalgesia in a dose-dependent manner when applied subcutaneously. Concentrations of NaHS used were 50 (**A**), 250 (**B**) and 1000 (**C**) μM in a volume of 50 μL. The maximal effect was observed at a dose of 1000 μM. The longest duration was 1.5 hours at concentration of 1000 μM. Normal saline (NS) at the same volume was used as control. NS did not produce any effect on escape threshold (**D**). *p<0.05, compared with NS.

To determine whether H_2_S led to hyperalgesia, NaHS, an H_2_S donor, was injected into the right side of temporomandibular joint (TMJ) capsule. Three doses of NaHS (50, 250 and 1000 μM) were used in this study. Administration of NaHS produced a reduction of mechanical threshold for escape behavior in a dose-dependent manner (Figure 5). The maximal hyperalgesic effect was observed at the dose of 1000 μM. In addition, the time course of NaHS effect was also studied. The effect produced by 1000 μM NaHS started at 30 mins after injection and lasted for 2 hours and returned to baseline at 4 hours after injection (Figure 5C). Injection of NS into the right side of TMJ capsule did not alter the escape threshold of rats (Figure 5D).

## Discussion

Our study demonstrates for the first time that endogenous H_2_S producing enzyme cystathionine β-synthase (CBS) is abundantly expressed by trigeminal ganglion neurons and co-localized in Kv1.1 and Kv1.4 positive neurons (Figure 1). CBS and systathionine γ-lyase (CSE) are two important endogenous enzymes for generation of H_2_S in mammals, and have been found in many types of mammalian cells in the central nervous system as well as in the peripheral tissues [28-30]. We have previously reported that CBS was predominately expressed by small and medium sized dorsal root ganglion neurons and co-localized well with purinergic P2X3 receptors and TRPV1 in colon specific neurons, suggesting a role for CBS in visceral hypersensitivity [11]. In this study, we showed that administration of NaHS, which mimics CBS production of H_2_S, reduced mechanical escape threshold in a dose-dependent manner (Figure 5), indicating that endogenous CBS-H_2_S signaling pathway might be involved in the trigeminal nociceptive processing.

H_2_S, formed by these two enzymes, has been found to regulate key neuronal functions, including induction of long-term potentiation and modulation of NMDA receptor currents in the hippocampus under physiological conditions [31]. In addition, H_2_S donor NaHS enhanced the excitability of dorsal root ganglion neurons *in vitro*[11]. In this study, we provide direct evidence that H_2_S donor NaHS sensitized TG neurons. This conclusion is based on several findings shown in Figures 2 and 3. First, NaHS application led to a marked depolarization of RP of TG neurons (Figure 2B). Secondly, these neurons exhibited lower current thresholds for initiating an action potential (AP, Figure 2C) and lower AP thresholds (Figure 2D). Finally, these neurons had an enhanced firing frequency in response to a standardized current stimulation after NaHS application (Figure 3). Together with our previous report that H_2_S enhanced the neuronal excitability of colon-specific DRG neurons [32], the present study indicates that H_2_S modulates membrane properties of rat primary sensory neurons.

Another interesting finding is that NaHS application significantly suppressed *I*_K_ density in TG neurons (Figure 4). To the best of our knowledge, it is the first report that H_2_S play an inhibitory effect on voltage-gated potassium channels of TG neurons. At least 6 types of K^+^ currents have been detected in sensory neurons; including both rapidly inactivating, A-type K^+^ currents (*I*_A_) and slow-inactivating sustained (K-current; *I*_K_) [20,21], and several different isoforms of K^+^ currents with different kinetics, and their related protein subunits, have been identified in primary sensory neurons. These voltage-gated K^+^ (Kv) channels are important physiological regulators of membrane potentials, action potential shape and firing frequencies in nociceptive sensory neurons and often shown to be decreased during injury-induced hyperalgesia [33,34]. Since the opening of K^+^ channels leads to hyperpolarization of cell membrane and a consequent decrease in neuronal excitability [35], the reduction in *I*_K_ density induced by NaHS may well contribute to the enhanced excitability of TG neurons. Although we do not know the intracellular pathway(s) for these effects of H_2_S, the acute inhibition of channel activity would likely explain these effects. However, other possibilities such as regulation of protein trafficking or endocytosis cannot be excluded. In addition, amplification of TRPV1 currents by H_2_S occurs via releasing tachykinins [36], and if this is also the pathway for H_2_S’s modulation of *I*_K_, then H_2_S acts on different receptors to activate separate intracellular pathways that converge on common targets. Their combined actions in the setting of peripheral tissue injury or inflammation probably ensures a strong, acute and prolonged response in nociceptor firing. When added to the amplified response of TRPV1 or purinergic receptors caused by H_2_S, and possibly others, e.g., nerve growth factors, the net effect will integrate a heightened transduction of nociceptive stimuli with reduced threshold and elevated repetitive firing to produce a powerful sensitization of TG nociceptors. Of note is that we do not know whether extracellular or intracellular or both sides of H_2_S mediate the modulation of potassium channels since H2S are membrane permeable. Future studies are needed to investigate the detailed mechanisms.

In addition to the effect of *I*_K_, the contribution of other K^+^ channels to the enhanced excitability of TG neurons has also to be considered. Transient K^+^ currents (*I*_A_) have been reported to be one of the major players in control of neuronal excitability [37]. In this study, however, NaHS incubation had no effect on *I*_A_, indicating a specific effect of H_2_S. TG neurons after NaHS application depolarized resting membrane potentials without changes in membrane input resistance. Changes in RP may be mediated by modulation of hyperpolarization-activated cation current (*I*_H_; an inwardly rectifying current) [38] or leakage current (*I*_L_; an outward resting current) [39]. However, the depolarization of RP in our study is unlikely due to an increase in *I*_H_, as this would be expected to result in a decrease in input resistance. Thus, depolarization of RPs in TG neurons after NaHS application may likely have resulted from a decrease in *I*_L_. However, in our experiments, when the RPs of TG neurons were corrected to the normal level (i.e., -55 mV) after NaHS application, the rheobase and cell spike frequency were not significantly changed compared with those before the correction of RPs (data not shown). Thus, even if there were changes in *I*_L_, they had a minimal effect on spike frequency and current threshold in this study. At this time we cannot rule out the possibility that H_2_S modulates other voltage-gated ion channels, including Na_V_ and the various Ca^2+^ channels. Previous studies have shown that NaHS/H_2_S activates or sensitizes Cav3.2 T-type Ca^2+^ channels expressed in the primary afferents [8] and TRPV1 and TRPA1 in nonvascular smooth muscles [7]. H_2_S is now thought to join the set of agents that enhance excitability; it too enhances TRPV1 currents and suppresses I_K_. These combined changes in the activities of different ion channels will have a strong, possibly synergistic effect in enhancing excitability, particularly for inducing repetitive firings to produce a powerful sensitization of TG nociceptors under pathophysiological conditions such as TMJ inflammation.

In conclusion, our data demonstrate that H_2_S enhanced the neuronal excitability and suppressed the I_A_ potassium current density, indicating that CBS-H_2_S signaling pathways may play a role in trigeminal physiology and pathophysiology. Previous studies in different trigeminal nerve inflammation/injury models suggested that the hyperexcitability of primary afferent neurons contributes to the hyperalgesia and allodynia. Further experiments are warranted to determine changes in CBS-H_2_S signaling under chronic pain conditions such as temporomandibular joint and related disorders.

## Competing interests

The authors declare that they have no competing interests.

## Authors’ contributions

All authors have read and approved the final manuscript. XMF and YLZ designed and performed the experiments, helped to interpret the data; XWM, FHQ and WC performed the experiments, analyzed the data and prepared the figures; XHJ coordinated the project, wrote the manuscript; GYX designed and supervised the experiments, and edited the manuscript.
